# Molecular Cloning and Characterization of the 5′-Flanking Regulatory Region of the Carbonic Anhydrase Nacrein Gene of the Pearl Oyster *Pinctada fucata* and Its Expression

**DOI:** 10.1007/s10528-012-9510-8

**Published:** 2012-05-10

**Authors:** Tomoyuki Miyashita, Akiko Takami, Ryosuke Takagi

**Affiliations:** Department Genetic Engineering, Faculty of Biology-Oriented Science and Technology, Kinki University, 930 Nishimitani, Kinokawa, Wakayama 649-6493 Japan

**Keywords:** Carbonic anhydrase, Nacrein, *Pinctada fucata*, Biomineralization, Promoter, C-jun

## Abstract

The carbonic anhydrase nacrein participates in the formation of the nacreous or prismatic layer of *Pinctada fucata*. We isolated a genomic clone containing the nacrein gene and cloned the 5′-flanking region. Within the 1336 bp 5′ flanking region, we identified putative cis-acting elements, including the TATA box (TATAAAA) at −82 bp, and AP1 (−819 bp) and Oct-1 (−1244 bp) binding sites. In addition to the mantle, the nacrein gene is also expressed in the adductor muscle, liver, and foot. These results showed that nacrein not only takes part in the formation of the hard tissue but also might be involved in acid–base balance, ion transport, and maintenance of ionic concentration. In vitro transcription experiments showed that the addition of human c-jun activates transcription from the nacrein promoter. This is the first report of a promoter from a gene that controls the formation of the hard tissue of mollusk shells.

## Introduction

Carbonic anhydrases (CA)(EC 4.2.1.1) are zinc-containing enzymes that catalyze the hydration of carbon dioxide and the dehydration of bicarbonate:

$$ {\text{CO}}_{2} + {\text{H}}_{2} {\text{O}} \Leftrightarrow {\text{HCO}}_{3}^{ - } + {\text{H}}^{ + } $$

CAs are widely distributed in all living things, from eukaryotes to prokaryotes, and play an important role in acid–base balance, ion transport, and maintenance of ionic concentration (Cameron 1979; Forster et al. 1986; Henry 1984, 1996). There are at least five distinct CA gene families: α, β, γ, δ, and ε (So et al. 2004). The similarity of the amino acid sequences among these proteins is not high. In the animal kingdom, previously reported CAs are composed of a single gene, the so-called α-carbonic anhydrase (Hewett-Emmett and Tashian 1996). In mammals, at least 15 different α-CA isozymes have been described; they can be divided into five subgroups by their localizations. Five families (CAI, CAII, CAIII, CAVII, and CAXIII) are cytosolic; others are membrane-bound (CAIV, CAIX, CAXII, CAXV, and CAXIV), mitochondrial (CAVA and CAVB), or secreted (CAVI). The three isoforms that lack CA activity are also known as CA-related proteins (CAVIII, CAX, and CAXI). These CAs show tissue-specific distribution (Tashian 1989; Pastorekova et al. 2004).

CA also participates in biomineralization and is an essential enzyme of calcification (Freeman 1960; Bensch 1984; Kakei and Nakahara 1996). In the biomineralization of bivalves, there is a correlation between CA activity and larval shell developmental steps (Medaković 2000). CA is an important marker of biomineralization during early shell formation in the European abalone *Haliotis tuberculata*, Linnaeus (Gaume et al. 2011). The mantle of the mollusk shell contains CA activity (Wilbur and Jodrey 1955; Yu et al. 2006; Medaković 2000). It is believed that the CA of mantle epithelia facilitates the secretion of HCO_3_− required for this calcification (Boer and Witteveen 1980).

Nacrein was first discovered as a carbonic anhydrase in the hard tissue of the bivalve pearl oyster *Pinctada fucata* (Miyamoto et al. 1996; Miyashita et al. 2002). Nacrein is composed of two functional domains: a carbonic anhydrase domain and the Gly-Xaa-Asn (Xaa = Asp, Asn, or Glu) repeat (Miyamoto et al. 1996). Homologs of nacrein were subsequently discovered: the carbonic anhydrase termed N66 (Kono et al. 2000) from *Pinctada maxim* and a carbonic anhydrase of the gastropod *Turbo marmoratus* (Miyamoto et al. 2003). The amino acid sequence of the repeat domain of nacrein and N66 are similar, except for a long NG repeat in N66. On the other hand, most of the repeat domain of *Turbo marmoratus* enzyme is composed of an NG repeat.

In vitro experiments showed that N66 strongly inhibited the crystallization of CaCO_3_ in the free state (Kono et al. 2000). In nacrein, the repeat domain participates in the inhibition of the calcium carbonate precipitation and crystal growth (Miyamoto et al. 2005).

In addition to the carbonic anhydrases mentioned above, there are some reports of carbonic anhydrases that are involved in hard tissue formation of invertebrates. Proteins were extracted with 0.5 M EDTA from the organic matrix spicules of the alcyonarian, *Synularia polydactyla*, and it is reported that two proteins (83 and 63 kDa) among them had carbonic anhydrase activity and calcium binding capability (Rahman et al. 2005). Calcium-binding carbonic anhydrases were identified from the nacre-soluble matrix in the freshwater mussel *Unio pictorum* (Marie et al. 2008). It is speculated that this enzyme might participate in the calcification process and may have a structure similar to that of nacrein.

The 1.3 kbp 5′ flanking sequence of the human CAII gene has strong promoter activity in renal tubular cells, and this region contains a putative transcription factor AP1 binding element (Lai et al. 1998). Carbonic anhydrase II (CAII) is essential for osteoclast activity. CAII in osteoclasts is transcriptionally up-regulated by a c-Fos/AP-1 transcription factor (David et al. 2001). Furthermore, the promoter of a tumor-associated isoenzyme MN/CAIX (MN) is also activated by AP1 (Kaluzova et al. 2001).

In the present study, to characterize the 5′-flanking transcriptional regulatory region, we isolated and sequenced the 5′-flanking region of nacrein. We performed in vitro transcription using HeLa nuclear extracts, which suggested that the addition of human c-jun activates transcription from the nacrein promoter. This is the first report of the isolation of a promoter from a gene that controls the formation of the hard tissue of mollusk shells.

## Materials and Methods

### Materials

Recombinant human c-jun was purchased from Promega. Pearl oyster (*P. fucata*) specimens were obtained from Gokasho Bay (Mie Prefecture, Japan) in August 2006.

### Construction of Genomic Library and Cloning of Nacrein

The construction of the genomic library and the cloning of genomic clones of nacrein were performed essentially as described previously (Miyashita et al. 2008), with the following modifications. Hybridization was performed at 50 °C, and the filters were washed twice with 0.1× SSC (standard saline citrate), 1 % SDS, for 10 min at room temperature, before being washed twice with 0.1× SSC, 1 % SDS, for 20 min at 50 °C.

### DNA Sequencing

DNA sequencing was performed as described previously (Miyashita et al. 2000).

### RNA Purification and Northern Hybridization

RNA was isolated from various tissues of *Pinctada fucata* (adductor muscle, outer edge of the mantle, inner part of the mantle, gill, foot, and liver) using the QuickPrep Total RNA Extraction Kit (Amersham Biosciences, England) according to the manufacturer’s instructions.

RNA samples (20 μg per lane) were separated by electrophoresis through a 15 % agarose gel containing 0.66 M formaldehyde and were then transferred to positively charged nylon membranes (Boehringer Mannheim, Mannheim, Germany). The membrane was hybridized with a nacrein cDNA of approximately 2.3 kbp or human β-actin cDNA and labeled with digoxigenin-dUTP by the random primer method (Boehringer Mannheim). Hybridization and detection were performed as described previously (Miyashita et al. 2008).

### Isolation of the 5′-Flanking Region

Genomic phage clone D was digested with *Not*I and *Eco*RI. The digested DNA was subjected to agarose gel electrophoresis and Southern blotting. The filter was washed with 6× SSC and then hybridized with an 82 bp *Not*I-*Dra*I fragment of 5′-terminus nacrein cDNA (Miyamoto et al. 1996) that was labeled with digoxigenin-dUTP by a random primer procedure (Boehringer Mannheim). The conditions for hybridization and detection of labeled probes were as described previously (Miyashita et al. 2008). A DNA fragment of approximately 1.9 kbp was visualized using a BCIP/NBT reagent. This fragment was purified from the agarose gel and cloned into the *Not*I and *Eco*RI sites of pBluescript SK sequencing vector, which was designated pBDEN78.

*Bgl*II digested the 1.9 kbp fragment into two fragments of 1.0 and 0.9 kbp. These fragments were cloned into the *Not*I-*Bam*HI and the *Bam*HI-*Eco*RI sites, respectively, of the pBluescript SK sequencing vector and sequenced. The 1.0 kbp fragment contained the 5′-flanking region. The 0.9-kbp fragment contained 0.4 kbp of the 3′ portion of the 5′-flanking region, 0.2 kbp of the first exon, and 0.3 kbp of the 5′ portion of the first intron.

### Plasmid Construction for In Vitro Transcription

The pBDEN78 has two *Eco*RV sites, one in the 5′ region of the 1.9 kbp genomic fragment and the other in the multiple cloning site. The length of the *Eco*RV fragment is about 1.6 kbp. The 1.6 kbp *Eco*RV fragment was cloned into the *Eco*RV site of the pBluescript SKII(+) vector, which was designated pBDEV4. The plasmid pBDEV4 has two *Hin*cII sites. To remove the putative AP1 binding site, it was necessary to remove the *Hin*cII site between the *Eco*RV and *Xho*I sites. Therefore, plasmid pBDEV4 was digested with *Sal*I and *Xho*I followed by self-ligation. The putative AP1 binding site is located between the *Sma*I and *Hin*cII sites. The *Sma*I site is in the multiple cloning site, and the *Hin*cII site is just to the 3′ side of the putative AP1 binding site. The plasmid pBDEV4 was digested with *Sma*I and *Hin*cII followed by self-ligation. The plasmid with the putative AP1 binding site deleted was designated pBDDS1.

### In Vitro Transcription and Detection

The in vitro transcriptions were performed by the following modified protocol of HeLaScribe nuclear extract in vitro transcription system, provided by the manufacturer (Promega). DIG RNA Labeling mix (rNTP: Boehringer Mannheim) was used to label run-off transcripts. Reaction mixtures of 12.5 μl were prepared by combining 1.5 μl HeLaScribe nuclear extract (58.2 μg) with 1.0 μl DIG RNA labeling mix (10×), 0.75 μl 50 mM MgCl_2_, 2.25 μl H_2_O, and 1.0 μl linearized nacrein promoter/5′-flanking region (50 ng/ml) as a template. The reactions were initiated by the addition of 4.0 μl HeLa extract and incubated at 30 °C for 1 h. Reactions were terminated by adding 87.5 μl Stop solution. After phenol/chloroform treatment, RNA transcripts were collected by ethanol precipitation. The precipitate was dissolved in water.

Transcripts were separated by 1.2 % agarose gel electrophoresis and then transferred to a nylon membrane (Boehringer Mannheim) by Northern blotting. After baking at 80 °C for 2 h, the filters were visualized with BCIP/NBT reagent, according to the manufacturer’s instructions (Boehringer Mannheim).

## Results

### Isolation of Genomic Clones and Analysis of the Genomic DNA Fragment

A library of approximately 5 × 10^5^ lambda FIX II phages was screened by plaque hybridization using the nacrein cDNA as the probe. Five positive plaques were isolated and designated clones A, B, C, D, and E. Restriction analysis showed that clones B, C, and D were identical (data not shown). The same analysis showed that clones A and E were different (data not shown). The length of the genomic fragment of clone D was longer than that of clone A. Moreover, although the 5′-flanking region was the same length in clones A and D, the breeding ratio of clone D was higher than that of clone A. Therefore, clone D was used for analysis.

Clone D was amplified and the DNA was purified and sequenced. Sequence analysis of the insert of clone D showed that it covers the region beginning about 1.4 kbp upstream from the putative transcription start site of the nacrein gene and ends at the *Mbo*I restriction site within the fifth intron of the nacrein gene (Miyashita et al. unpublished result). The DNA was completely digested with *Eco*RI and *Not*I. After agarose gel electrophoresis, Southern blotting was performed, followed by hybridization with the 82 bp *Not*I-*Dra*I fragment of the 5′ end of the nacrein cDNA (Miyamoto et al. 1996). An *Eco*RI-*Not*I fragment of approximately 1.9 kbp hybridized with the probe. The fragment was cloned into pBluescript SK sequencing vector, which was designated pBDEN78. The restriction enzyme and sequencing analysis suggested that this fragment contained the 5′-flanking region, the first exon, and the 5′ terminal portion of the first intron.

### Characterization of 5′-Flanking Region and Assay of Promoter Activity

**Fig. 1:**
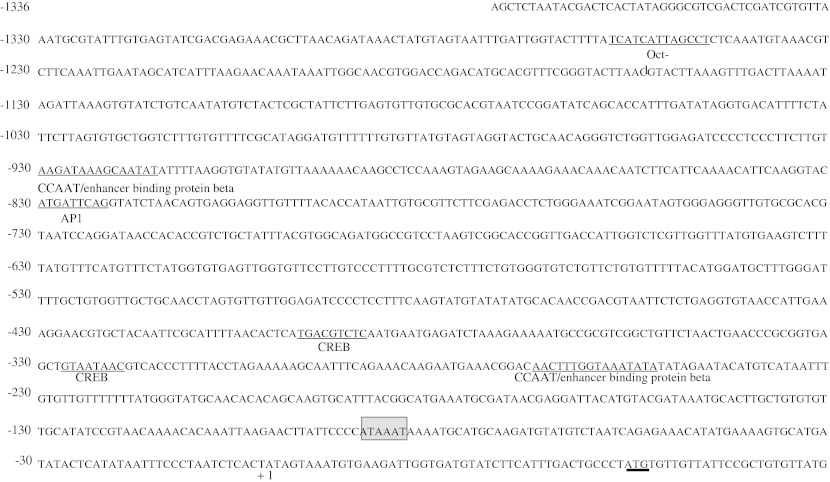
DNA sequence of the nacrein promoter region. Putative transcription factor binding elements are *underlined*, with a *bold underline* at the translational initiation codon (ATG). The TATA *box* is *shaded gray*. Nucleotides in this region are numbered with reference to the base position at the putative transcriptional start site (+1), located at the 5′ terminal nucleotide of the nacrein cDNA. GenBank accession nos.: D83523 for the nacrein cDNA, AB274024 for the promoter region, AB534918 for the first exon

The 1336-bp sequence of the 5′-flanking region of the nacrein gene was analyzed (Fig. 1). The putative cis-acting elements within the analyzed region include the TATA box located at −82 bp, the AP1 binding site at −819 bp, and the Oct-1 binding site at −1244 bp. Possible transcription factor CCAAT/enhancer-binding protein β (C/E BPβ) binding sites were also identified at −283 and −916 bp upstream of the putative transcriptional start site.

**Fig. 2:**
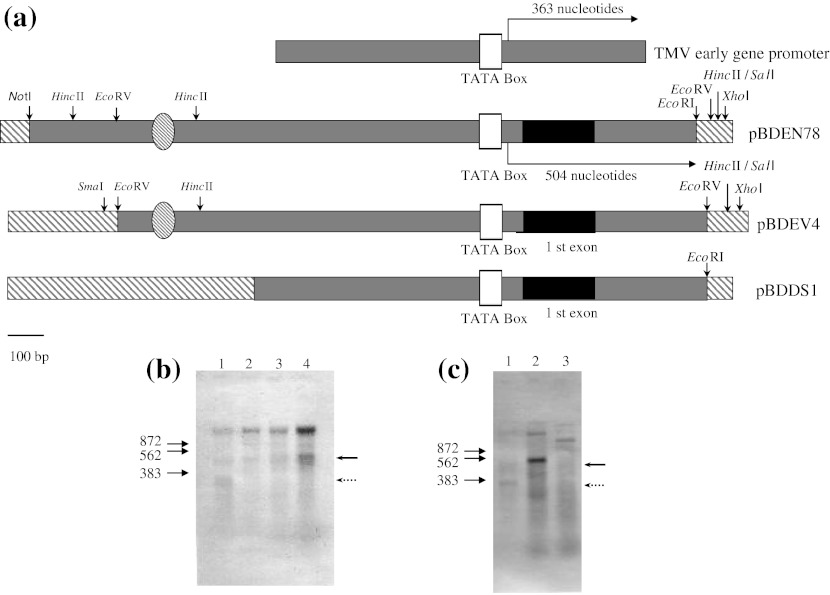
In vitro transcription from the nacrein gene promoter. **a** Schematic representation of the DNA template used for the in vitro transcription assays. *Gray bar* genomic DNA regions, *black bar* first exon, *white box* TATA box, *striped bar* pBluescript SK vector, *striped oval* putative AP1 binding site, *vertical arrow* position of the restriction enzyme site indicated. The putative transcription start site is at +1 and is equivalent to the 5′ terminus of the cDNA. **b** In vitro transcription of CMV early gene promoter (*lane 1*) and pBDEN78 (*lanes 2–4*), used as the template. Amount of recombinant human c-Jun: 0.5 μg in *lane 2*, 1 μg in *lane 3*, 2 μg in *lane 4*. **c** In vitro transcription of CMV early gene promoter (*lane 1*) and pBDEN78 (*lane 2*) and pBDDS1 (*lane 3*) used as templates, with 2 μg recombinant human c-Jun added to lanes 2 and 3. *Arrows* on *left* of gels indicate positions of the RNA size markers. *Solid arrow* on *right* indicates a transcript of about 500 nucleotides; *dotted arrow* indicates a transcript of about 360 nucleotides

The promoter activity was then studied using an in vitro transcription system based on HeLa cell nuclear extract (Fig. 2). Plasmid pBDEN78, containing sequences from +504 to −1336 bp, was linearized with *Eco*RI at +504 and used as the template for run-off in vitro transcription. If the TATA box is functional, a transcript of about 500 nucleotides, which is equivalent to the length from the +504 *Eco*RI site in the first intron to the putative transcription start site at +1, should have been detectable, and indeed it was (Fig. 2). This result showed that this region, including the putative TATA box, functions as the nacrein promoter. We used a 1.2-kbp DNA fragment containing a TMV early gene promoter as a control. Because the promoter is located about 363 bp upstream from the 3′ terminus, a transcript of about 360 nucleotides should be produced; again, the expected transcript was observed (Fig. 2).

To study the effects of c-jun on nacrein promoter activity, we added purified human c-jun protein to the in vitro transcription system based on HeLa cell nuclear extract. The addition of human c-jun protein activated transcription from the nacrein promoter, and the amount of the 500 nt transcript increased with the amount of added c-jun (Fig. 2). To test the possibility that c-jun functions through the putative AP1 binding site located at −819, we constructed plasmid pBDDS1, which lacks the putative AP1 binding site. We performed in vitro transcription experiments using both pBDEN78 and pBDDS1 as templates and adding c-jun to stimulate transcription. Although the 500-nt transcript was observed when using pBDEN78, it was not observed when pBDDS1 was used as a template (Fig. 2).

In these experiments, the longest transcript in each lane corresponds to the product transcribed from the entire length of the template DNA by RNA polymerase II in the nuclear extracts. These products are nonspecific, and this phenomenon is often observed in the in vitro run-off transcription system.

### Expression of the Nacrein Gene

**Fig. 3:**
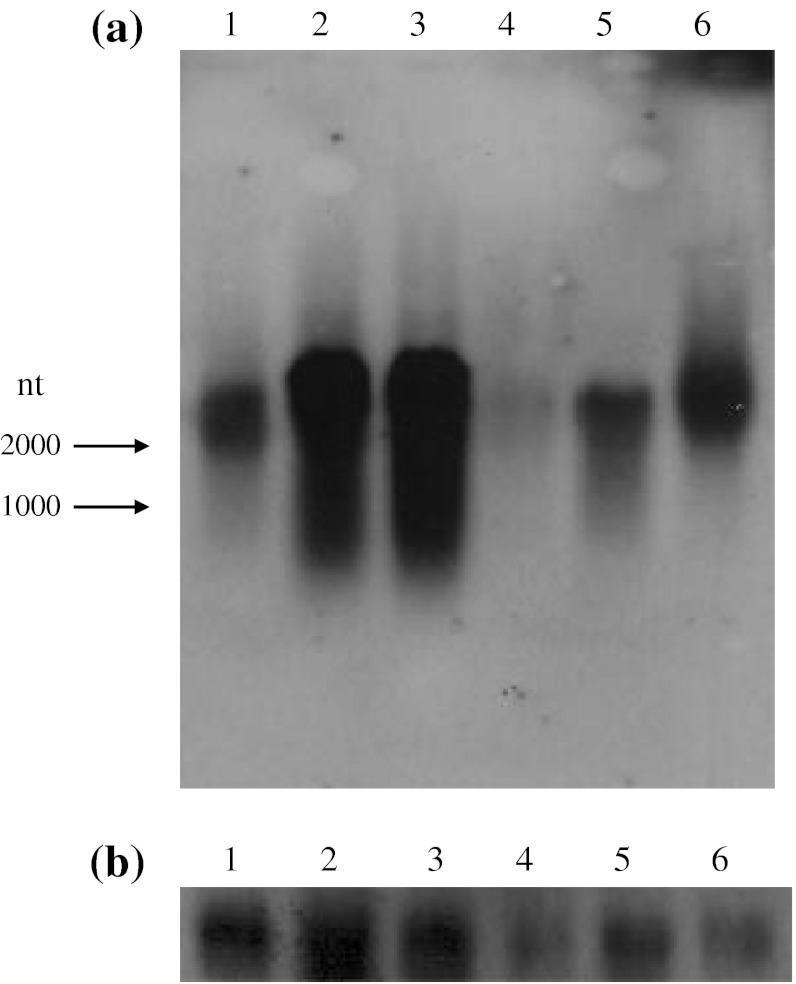
Northern blot analysis of the transcripts. **a** *Pinctada fucata* *nacrein* gene. **b** β-*actin* gene. Tissues: *Lane 1* adductor muscle, *2* outer edge of the mantle, *3* inner part of the mantle, *4* gill, *5* foot, *6* liver. The nacrein cDNA and the human β-actin cDNA were used as probes. *Arrows* indicate size markers (1000 and 2000 nucleotides)

Real-time quantitative PCR analysis and in situ hybridization revealed that the nacrein mRNA was expressed in the mantle edge, corresponding to the prismatic layer formation, and in the inner part of the mantle tissue, corresponding to the nacreous aragonite shell layer (Takeuchi and Endo 2006; Miyamoto et al. 2005). To examine the tissue-specific expression of nacrein mRNA, we performed Northern blot analysis in various tissues of *Pinctada fucata* (Fig. 3). The nacrein gene was actively transcribed in the inner part and the edge of the mantle, and also in the adductor muscle, the liver, and the foot at high levels. Expression was also observed in the gill, though at a low level. The expression of β-actin mRNA was used as a control to show that all samples contained intact RNA.

## Discussion

Nacrein was the first carbonic anhydrase to be discovered in the nacreous layer of *Pinctada fucata* (Miyamoto et al. 1996). It was later shown to be expressed in the prismatic layer (Miyashita et al. 2002). Nacrein contains two functional domains, a carbonic anhydrase and a Gly-Xaa-Asn repeat domain (Xaa = Asp, Asn, or Glu). N66 is a homolog of nacrein of *Pinctada maxima*, and in vitro experiments have shown that N66 strongly inhibits the crystallization of CaCO_3_ in the free state (Kono et al. 2000). Detailed analysis showed that nacrein prevents crystal formation via an interaction between the Gly-Xaa-Asn repeat and certain crystal faces (Miyamoto et al. 2005).

Nacrein mRNA is transcribed actively in the mantle epithelium (Miyamoto et al. 2005; Takeuchi and Endo 2006). In this study, Northern blot analysis was performed for other tissues in addition to the mantle epithelium, and the nacrein gene was demonstrated to be transcribed in the adductor muscle, the liver, and the foot at high levels. The expression was also observed in the gill, though at a low level. Recently, the correlation between the transcription of the chicken type II procollagen gene and the translation of the mRNA was reported. In this report, although the mRNA was expressed in multiple tissues, the protein was only detected in chondrogenic cartilage, vitreous body, and cornea (Caixia et al. 2006). Thus, the presence of the mRNA in the different tissues does not mean that the protein is necessarily expressed there. Nevertheless, if nacrein is expressed in these tissues, it might play an important role in acid–base balance, ion transport, or maintenance of ionic concentration in the adductor muscle and gill.

Carbonic anhydrase II is necessary for osteoclast differentiation, and its transcription is activated by c-Fos/AP-1 (David et al. 2001). A tumor-associated carbonic anhydrase MN/CA9 (MN) gene is also activated by transcription factor AP1 (Kaluzova et al. 2001). The 5′-flanking region of the nacrein gene contains a putative AP1 binding site, in addition to a TATA box (ATAAAT) and possible CCAAT box (CCACT). To examine the promoter activity, we tried to isolate nuclei from the mantle of *Pinctada fucata* to prepare nuclear extracts for in vitro transcription. Unfortunately, we failed to isolate good quality nuclei. This failure may have been due to the special structure of the mantle. The mantle is a complex organ composed of an epithelium layer covering the mantle surface, muscle, connective tissue, and nerve fibers (Waller 1980). It is possible that the large amount of cytoskeletal protein and the pigments in the mantle affected the separation of nuclei.

HeLa cells are epithelial cells derived from cervical cancer cells (Scherer et al. 1953). We used HeLa nuclear extracts for in vitro transcription as a substitute for *Pinctada fucata* nuclear extracts. In eukaryotic cells, promoters have been proven to be functional across species. For example, the TATA sequence of the octopine-type T-DNA (TC7) is functional in the in vitro transcription system using HeLa nuclear extracts (Yamaguchi et al. 1998). Promoters from *Drosophila* heat shock proteins and cytomegalovirus are capable of driving the expression of introduced genes in embryos of the oyster *Crassostrea gigas* (Cadoret et al. 1997).

We examined the promoter activity of the nacrein gene in an in vitro transcription system using HeLa nuclear extracts. The addition of human c-jun protein to the in vitro transcription system increased the amount of transcript from the nacrein promoter. The amount of the 500-nt transcript (corresponding to the region between a putative transcription start site to an *Eco*RI site in the first intron) increased dependent on the amount of c-jun added. Deletion analysis showed that c-jun functions through the putative AP1 binding site located at −830 bp. These results suggest that the AP1 binding site and the TATA box of the nacrein gene are functional in an in vitro transcription system using Hela nuclear extracts.

C-jun functions as a transcription factor and forms a homo dimer or a hetero dimer with c-Fos, to form the transcription factor AP1 (Kerppola and Curran 1995; Angel and Karin 1991). Accordingly, the results of the in vitro transcription experiments suggested that transcription of the nacrein gene is activated through transcription factor c-jun, which forms a homo dimer. It is expected that c-jun functions as a hetero dimer with c-Fos in vivo. Our results also show that this in vitro transcription system is functional between vertebrate and invertebrate animals.

In the mantle epithelium, nacrein mRNA is actively transcribed and translated. After translation, the signal peptide causes the secretion of nacrein from the mantle epithelium. The synergistic interaction between the promoter and a transcriptional regulatory element, such as an enhancer, regulates this transcription. The nacrein promoter is the first promoter from a gene that controls hard tissue formation of mollusk shells to be reported.
